# Management of Gingival Recession of the Maxillary Left Canine Using a Coronally Advanced Buccal Flap and Palatal Connective Tissue Graft

**DOI:** 10.7759/cureus.107652

**Published:** 2026-04-24

**Authors:** Richard M Cavero, Jhonny L Gonzalez Ortega, Ines A Camejo

**Affiliations:** 1 General Practice, Metro Dental Associates, New Jersey, USA; 2 Dentistry, Boston University Henry Goldman Dental School of Dental Medicine, Boston, USA; 3 Dentistry, Smile and Implant Center of Rockland, New York, USA

**Keywords:** buccal flap, connective tissue graft, coronally advanced flap, esthetic zone, gingival recession, palatal graft, periodontal plastic surgery, root coverage

## Abstract

Gingival recession in the anterior maxillary region presents both functional and esthetic concerns, particularly when it affects the smile zone. Root exposure may lead to dentin hypersensitivity, plaque accumulation, and compromised gingival harmony. Periodontal plastic surgery techniques have been developed to restore gingival coverage and improve soft tissue thickness, demonstrating predictable results with generally favorable and well-documented outcomes.

This case report describes the treatment of localized gingival recession affecting the maxillary left canine (tooth #11, Universal Numbering System) using a coronally advanced buccal flap combined with a subepithelial connective tissue graft harvested from the palate. After administration of local anesthesia (2% lidocaine with 1:100,000 epinephrine), a partial-thickness coronally advanced buccal flap was carefully elevated at the recipient site, and the exposed root surface was thoroughly debrided. A subepithelial connective tissue graft harvested from the palatal donor site was trimmed, positioned over the recession defect, and stabilized with 3-0 chromic gut absorbable sutures. The buccal flap was then coronally advanced to fully cover the graft and secured with interrupted 3-0 chromic gut sutures to achieve tension-free closure.

Postoperative healing progressed without complications. Follow-up evaluation demonstrated improved gingival thickness and satisfactory root coverage, with a reduction of gingival recession from approximately 3 mm preoperatively to complete root coverage at the treated site. Probing depth remained stable at 2 mm, and enhanced esthetic integration with the surrounding tissues was observed.

This approach appears to be a reliable technique in this case, demonstrating favorable clinical and esthetic outcomes for managing gingival recession in the anterior esthetic zone while improving soft tissue stability and patient satisfaction.

## Introduction

Gingival recession adversely affects both oral health and esthetic appearance, particularly when it occurs in the anterior maxillary region, commonly referred to as the smile zone. It is defined as the apical migration of the gingival margin relative to the cemento-enamel junction, resulting in exposure of the root surface. This condition may lead to dentin hypersensitivity, increased susceptibility to root caries, plaque accumulation, and compromised gingival esthetics [1].

The etiology of gingival recession is multifactorial [2]. It may include traumatic toothbrushing, periodontal inflammation, a thin periodontal phenotype, orthodontic tooth movement beyond the alveolar envelope, and anatomical factors such as tooth malposition or inadequate keratinized gingiva.

Periodontal plastic surgery techniques have been developed to correct mucogingival defects and achieve predictable root coverage. Among these approaches, the use of a subepithelial connective tissue graft combined with a coronally advanced flap has demonstrated highly favorable clinical outcomes. This technique improves gingival thickness, increases the width of keratinized tissue, and provides stable and esthetic root coverage results [3,4]. The subepithelial connective tissue graft technique was first described by Langer and Langer and remains one of the most widely accepted surgical procedures for the treatment of localized gingival recession defects due to its predictable outcomes and excellent color match with adjacent gingival tissues [5]. Long-term clinical studies have also demonstrated that coronally advanced flap procedures combined with connective tissue grafts provide stable periodontal outcomes and long-term root coverage success [6].

This case report describes the management of localized gingival recession affecting the maxillary left canine (tooth 11) using a coronally advanced buccal flap combined with a subepithelial connective tissue graft harvested from the palate. The surgical procedure and clinical outcomes are presented.

## Case presentation

**Figure 1:**
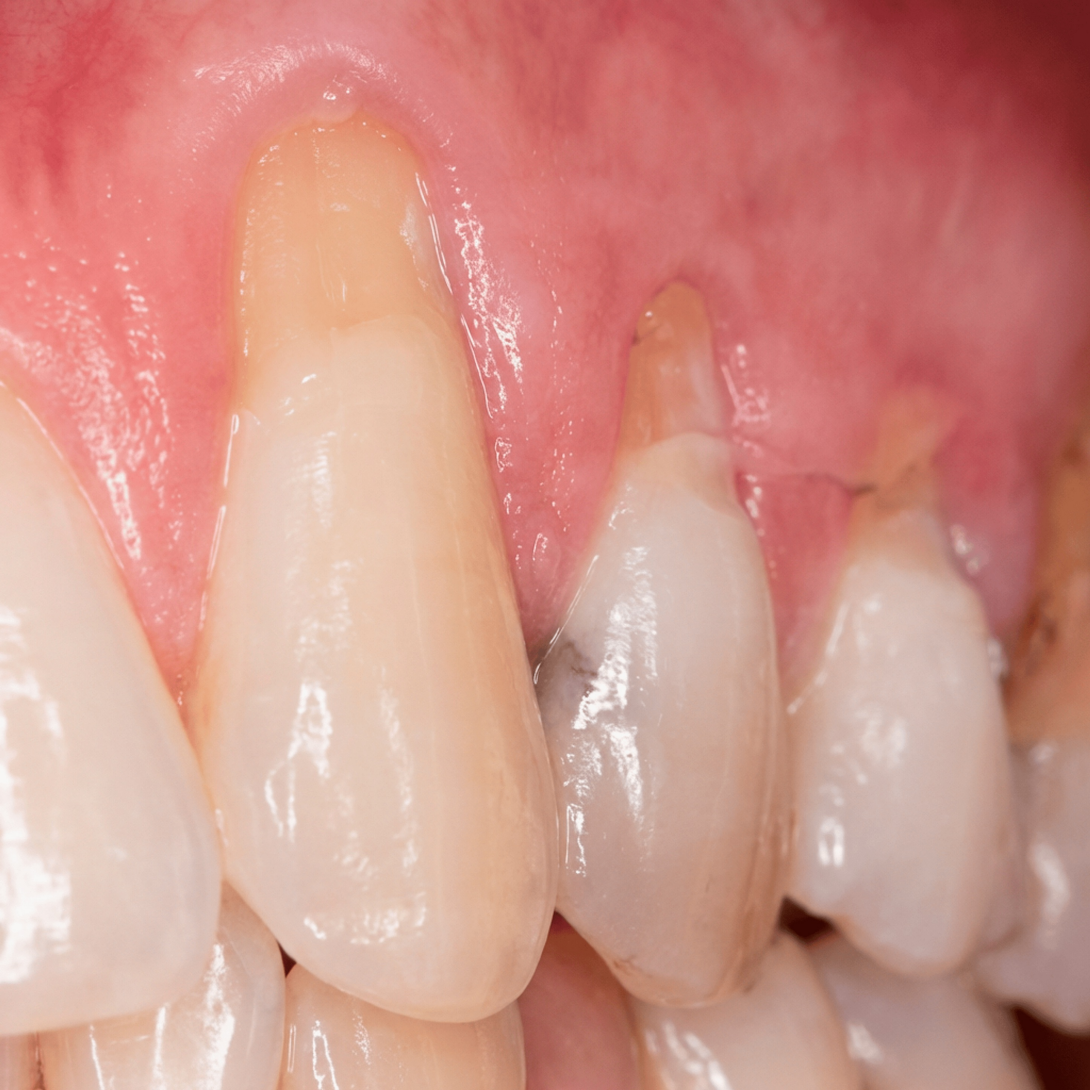
Clinical view of the gingival recession defect involving the maxillary left canine (tooth 11). The exposed root surface and apical displacement of the gingival margin are visible.

A 34-year-old male patient presented to the dental clinic with concerns regarding gingival recession affecting the maxillary left canine (tooth #11, Universal Numbering System). The patient reported dentin hypersensitivity and dissatisfaction with the esthetic appearance of the exposed root surface. The patient was systemically healthy and reported no significant medical history. Clinical examination revealed localized gingival recession measuring approximately 3 mm in depth and 2 mm in width, with visible root exposure and a thin gingival biotype in the affected area (Figure 1). The defect was classified as Miller Class I and Cairo RT1, indicating a favorable prognosis for complete root coverage [2,7].

**Figure 2:**
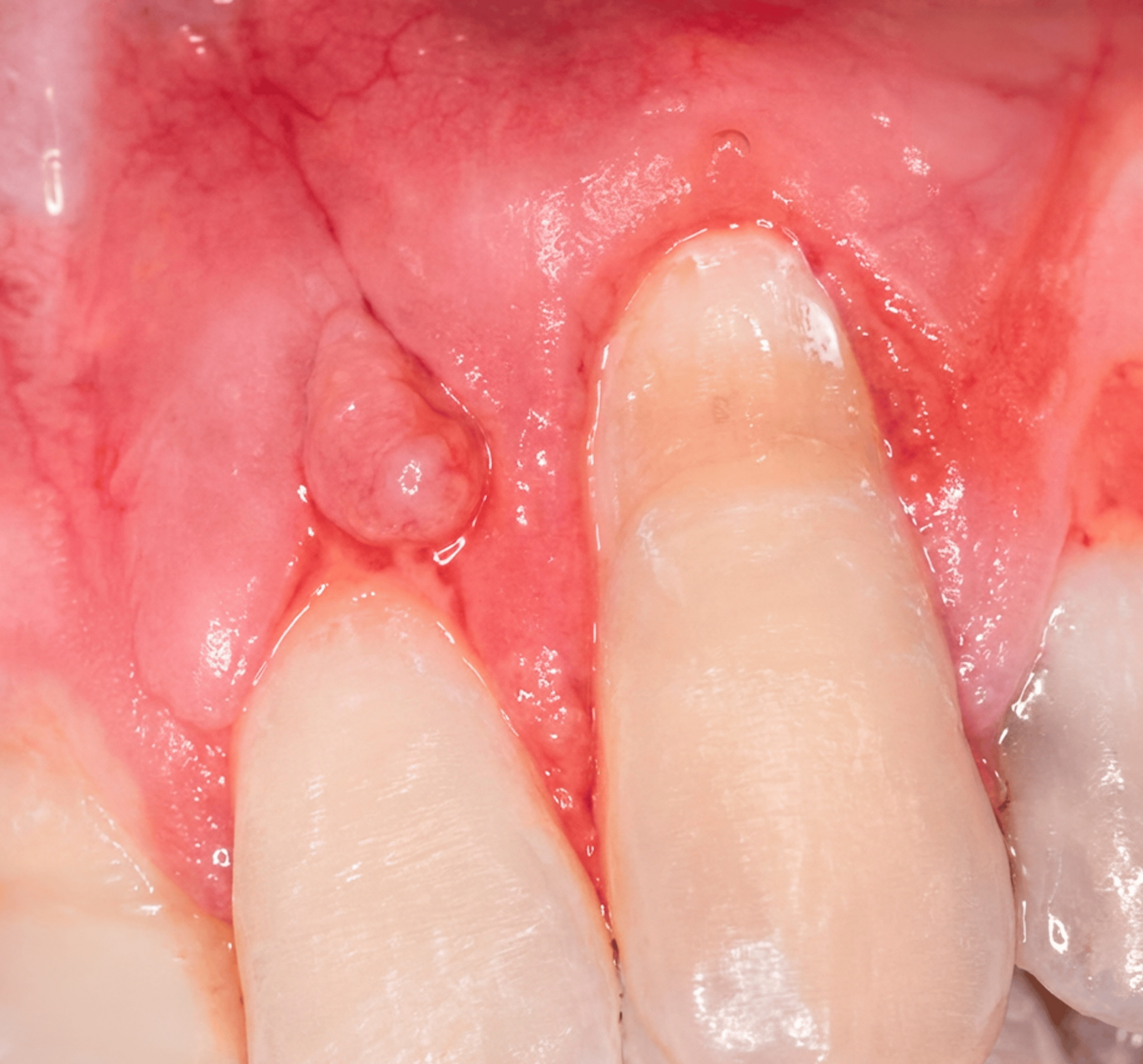
Preoperative clinical view of gingival recession affecting the maxillary left canine. The extent of the recession defect and gingival margin position are visible.

After clinical evaluation and treatment planning, periodontal plastic surgery was performed using a buccal flap approach combined with a subepithelial connective tissue graft harvested from the palatal donor site. Local anesthesia was administered using 2% lidocaine with epinephrine (1:100,000). A partial-thickness buccal flap was carefully elevated at the recipient site through horizontal and vertical releasing incisions to allow coronal repositioning of the soft tissue (Figure 2). The exposed root surface was thoroughly scaled and root planed to remove plaque and calculus deposits and to prepare the root surface for graft adaptation.

**Figure 3:**
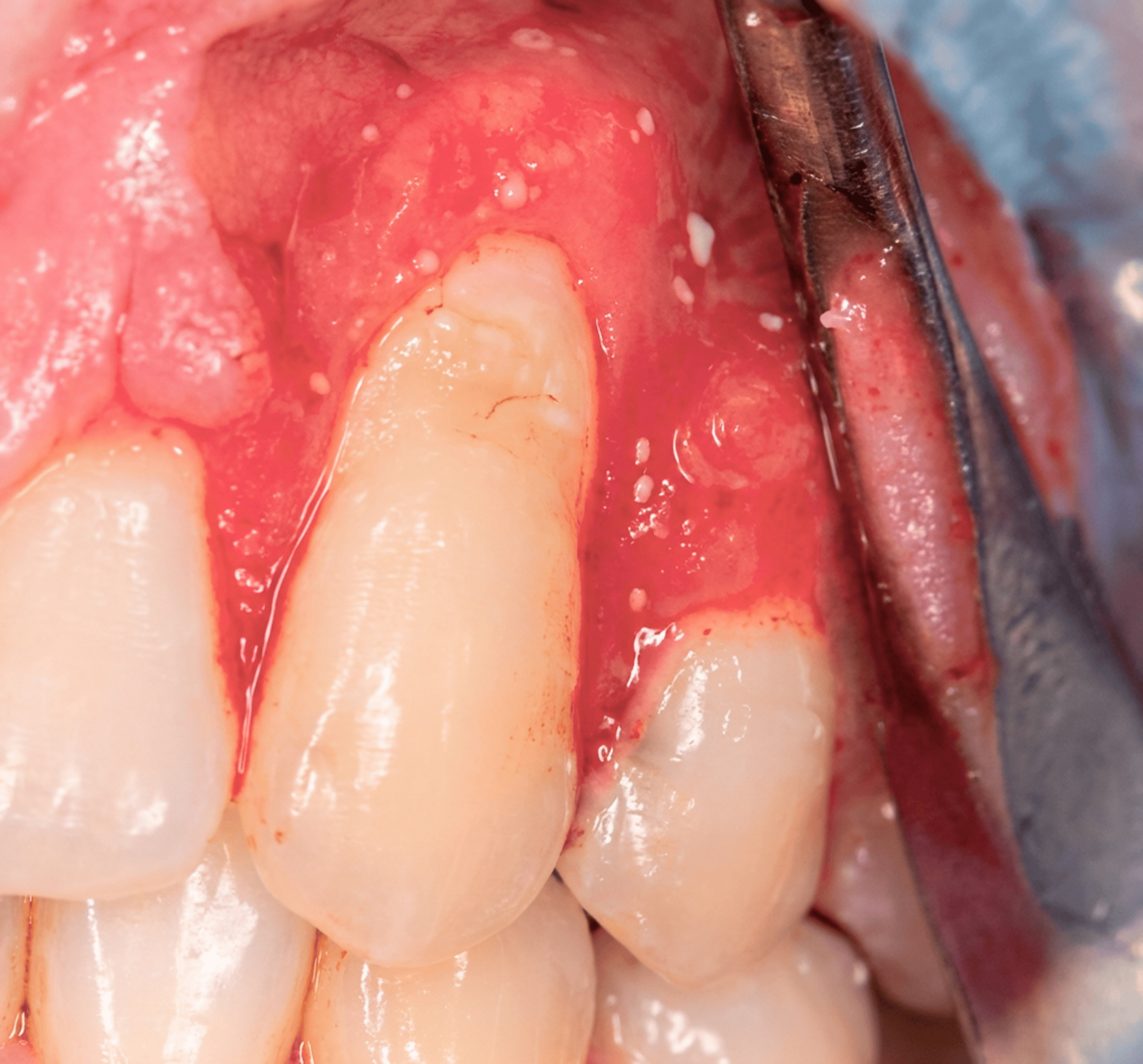
Intraoperative view showing elevation and mobilization of the buccal flap at the maxillary left canine (tooth 11) before coronal advancement. The surgical flap and exposed root surface are visible.

**Figure 4:**
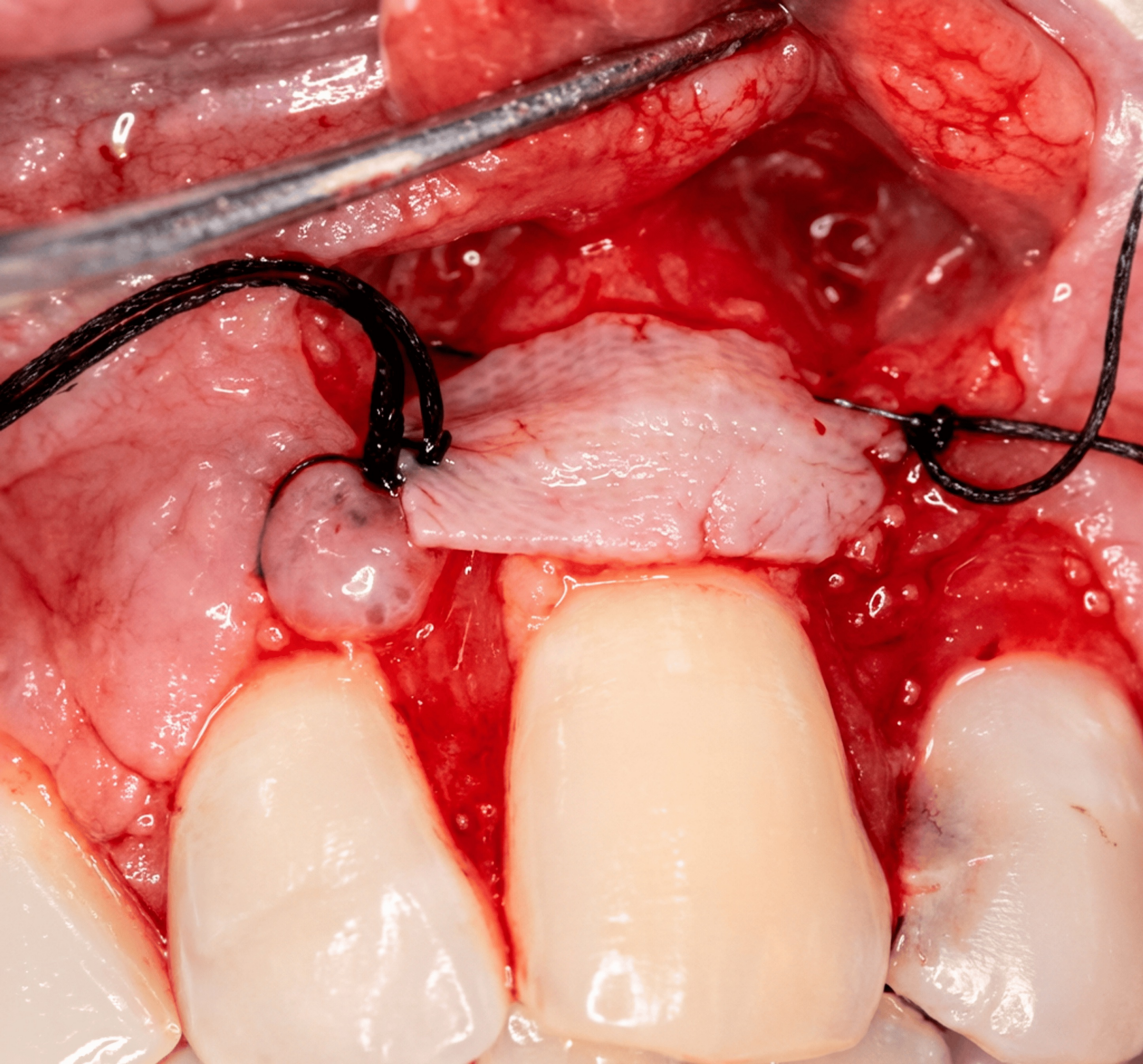
Intraoperative view showing placement of the subepithelial connective tissue graft over the exposed root surface of the maxillary left canine (tooth 11). The connective tissue graft is placed and stabilized with sutures at the recipient site.

**Figure 5:**
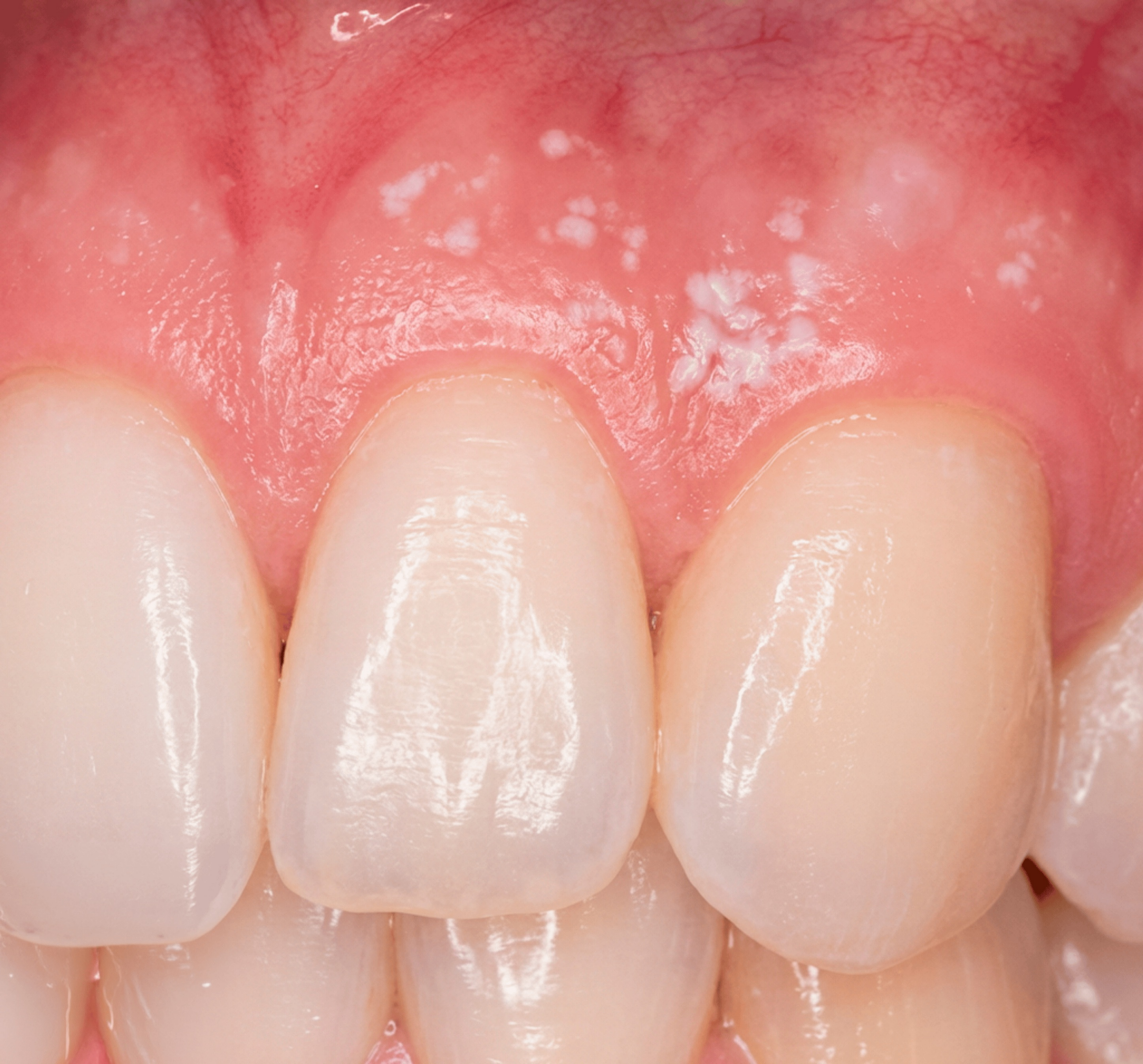
Postoperative clinical view showing successful root coverage of the maxillary left canine (tooth 11) following treatment with a coronally advanced buccal flap and subepithelial connective tissue graft. The improved gingival margin position and root coverage are visible.

A subepithelial connective tissue graft was harvested from the palatal donor site using a trap-door technique (Figure 3). The graft was carefully trimmed and positioned over the exposed root surface at the recipient site (Figure 4). The graft was stabilized with 3-0 chromic gut absorbable sutures to ensure adequate adaptation and immobility. Subsequently, the buccal flap was coronally advanced to completely cover the connective tissue graft and secured with interrupted 3-0 chromic gut sutures to achieve tension-free closure (Figure 5).

Postoperative instructions were provided, including avoidance of mechanical brushing in the surgical area for two weeks and use of 0.12% chlorhexidine mouth rinse twice daily (morning and evening) for one week. Healing progressed without complications. At three months follow-up, examination demonstrated improved gingival thickness, satisfactory root coverage, and enhanced esthetic integration with the surrounding tissues in the anterior maxillary region (Figure 5).

## Discussion

The management of gingival recession in the anterior maxillary region is particularly important due to the functional and esthetic concerns associated with root exposure. Patients with gingival recession often experience dentin hypersensitivity, increased plaque accumulation, and dissatisfaction with smile appearance. Periodontal plastic surgery techniques have been developed to address these mucogingival defects and achieve predictable root coverage outcomes [1].

The Cairo classification has been proposed as a contemporary system for the evaluation of gingival recession defects and provides a more precise assessment of interproximal attachment loss, thereby improving the prediction of root coverage outcomes [8].

Among the available surgical approaches, the subepithelial connective tissue graft combined with a coronally advanced flap is widely regarded as a predictable and effective technique for the treatment of localized gingival recession. This procedure improves soft tissue conditions and increases the width of the keratinized tissue, contributing to the long-term stability of the gingival margin [2]. The connective tissue graft technique, first described by Langer and Langer, has demonstrated high success rates and favorable esthetic integration with adjacent tissues, making it a widely used approach in periodontal plastic surgery [3].

Clinical studies have demonstrated favorable root coverage outcomes when connective tissue grafts are used in combination with coronally advanced flaps. These procedures also provide superior esthetic outcomes compared with free gingival grafts due to improved color match with surrounding tissues [5].

In the present case, the use of a coronally advanced buccal flap combined with a subepithelial connective tissue graft resulted in successful root coverage, increased gingival thickness, and improved esthetic integration. These findings are consistent with previously reported outcomes in the literature [6].

While the results of this case are encouraging, the findings should be interpreted with caution, given the limitations inherent to a single case report. Further studies with larger sample sizes and longer follow-up periods are necessary to confirm the generalizability and long-term effectiveness of this approach.

## Conclusions

The management of gingival recession in the anterior maxillary region requires predictable surgical techniques that restore both function and esthetics. In this case, the use of a coronally advanced buccal flap combined with a subepithelial connective tissue graft harvested from the palate resulted in successful root coverage, increased gingival thickness, and improved soft tissue stability. The procedure also enhanced the esthetic appearance of the treated area and reduced dentin hypersensitivity reported by the patient. These findings demonstrate the favorable clinical and esthetic outcomes achievable with this approach in a single case. Careful case selection, meticulous surgical technique, and appropriate postoperative care contributed to the positive outcomes observed. While encouraging, further studies with larger sample sizes are needed to confirm the generalizability of these results.
